# Efficacy and safety of migalastat in a Japanese population: a subgroup analysis of the ATTRACT study

**DOI:** 10.1007/s10157-019-01810-w

**Published:** 2019-12-30

**Authors:** Ichiei Narita, Toya Ohashi, Norio Sakai, Takashi Hamazaki, Nina Skuban, Jeffrey P. Castelli, Hjalmar Lagast, Jay A. Barth

**Affiliations:** 1 Division of Clinical Nephrology and Rheumatology, Niigata University Graduate School of Medical and Dental Sciences, Niigata, Japan; 2 Division of Gene Therapy, Research Center for Medical Sciences, The Jikei University School of Medicine, Tokyo, Japan; 3 Division of Health Science, Osaka University Graduate School of Medicine, Osaka, Japan; 4 Department of Pediatrics, Osaka City University Graduate School of Medicine, Osaka, Japan; 5 Amicus Therapeutics, Inc., 1 Cedar Brook Drive, Cranbury, NJ 08512 USA

**Keywords:** Fabry disease, Migalastat, Alpha-galactosidase, Pharmacologic chaperone, Mutation, Enzyme replacement therapy

## Abstract

**Background:**

Fabry disease is a progressive X-linked lysosomal disorder. In this subgroup analysis of the global phase III ATTRACT study, the efficacy and safety of oral migalastat, a pharmacologic chaperone, were investigated in Japanese patients with Fabry disease.

**Methods:**

Patients were randomly assigned to receive migalastat (150 mg every other day) or to continue biweekly enzyme replacement therapy infusions (ERT; agalsidase alfa 0.2 mg/kg or agalsidase beta 1.0 mg/kg) for 18 months followed by a 12-month open-label extension during which all patients received migalastat. End points included glomerular filtration rate (estimated and measured), left ventricular mass index (LVMi), composite clinical outcomes, leukocyte alpha-galactosidase A activity, plasma globotriaosylsphingosine (lyso-Gb_3_), and safety.

**Results:**

Data from 7 Japanese patients (migalastat, 5; ERT, 2), mean age 55 years, with high disease burden, were analyzed. All patients in the migalastat group completed the open-label comparison and extension periods. At 18 months, efficacy in the Japanese patient population was similar to that in the overall ATTRACT population. Migalastat treatment increased leukocyte alpha-galactosidase A activity, stabilized renal function, and decreased LVMi. Plasma lyso-Gb_3_ levels remained low and stable. Additionally, the long-term extension study showed that efficacy of migalastat was maintained for up to 48 months. Migalastat was safe and well tolerated in the Japanese patients, as in the overall ATTRACT population.

**Conclusion:**

Migalastat can be used to treat Japanese patients with Fabry disease with *GLA* mutations amenable to migalastat according to the dosage and administration approved in other countries.

**Trial registration numbers:**

ClinicalTrials.gov, NCT01218659 and NCT02194985.

## Introduction

Fabry disease is a rare progressive X-linked lysosomal disorder, in which mutations of the *GLA* gene impair the activity of the lysosomal enzyme alpha-galactosidase A (α-Gal A), resulting in a devastating condition [1]. In Fabry disease, accumulation of substrates of α-Gal A, such as globotriaosylceramide (GL-3) and globotriaosylsphingosine (lyso-Gb_3_), in various organs and tissues causes dysfunction that can lead to premature death. Cardiac complications are the primary cause of death in men and women both, although some data indicate that the primary cause of death is renal complications in men and cerebrovascular disease in women [2, 3]. The estimated prevalence of Fabry disease ranges between 1 in 476,000 and 1 in 117,000 worldwide, although its actual prevalence is thought to be higher [3]. In Japan, the estimated prevalence is 1 in 7000 newborns based on the results of neonatal screening [4].

Phenotypic expression of Fabry disease is highly variable. Initial symptoms of the classic disease generally appear in childhood, and symptoms progress if the condition is not treated [3]. Enzyme replacement therapy (ERT) with agalsidase alfa or agalsidase beta is the mainstay of treatment [5]. Results of previous clinical studies have shown efficacy and good tolerability of ERT in some Japanese patients with Fabry disease [6, 7]. However, several challenges remain, including infusion-associated reactions, reduced quality of life associated with lifelong parenteral treatment, and the decrease in efficacy after development of circulating antibodies to the enzyme [8, 9].

Migalastat is a low-molecular-weight iminosugar that can act as a pharmacologic chaperone by binding selectively and reversibly to the active site of specific mutant forms of α-Gal A, the genotype of which is referred to as amenable *GLA* mutations. Migalastat binds to mutant forms of α-Gal A in the endoplasmic reticulum and promotes trafficking to the lysosomes, increasing lysosomal enzyme activity [10–12]. The efficacy of migalastat has been verified in patients with amenable mutations [13]. Migalastat was discovered in Japan [14] and was approved for the treatment of patients with Fabry disease 16 years or older in Japan, [16] Australia, Europe, Israel, South Korea and Switzerland and in adult patients in Canada and the United States [10, 15].

A phase I pharmacokinetic study showed similar dose-proportional pharmacokinetics and a similar safety profile of migalastat in Japanese and non-Japanese populations [17]. The phase III ATTRACT study compared efficacy and safety of migalastat with ERT in patients with Fabry disease with amenable mutations who were previously treated with ERT [18]. During an 18-month treatment period, migalastat and ERT both had a similar effect on renal function. From baseline to month 18, the left ventricular mass index (LVMi) decreased significantly in the migalastat group, but there was no significant decrease in the ERT group [18]. We report the results of analyses performed in the Japanese subgroup, including data from the open-label extension study (OLE).

## Methods

### Patients and study design

ATTRACT was a global, open-label, randomized trial with a 30-month treatment period (18-month open-label comparison of migalastat with ERT, and 12-month OLE with migalastat; AT1001-012, ClinicalTrials.gov, NCT01218659) in patients with Fabry disease who were previously treated with ERT. Full methods are described in the primary paper [18]. Primary inclusion criteria were men and women between 16 and 74 years of age with Fabry disease and a migalastat-amenable *GLA* mutation, as detected by the preliminary human embryonic kidney (HEK) 293 cell-based assay [19]. Other inclusion criteria were initiation of ERT ≥ 12 months before the baseline visit, maintenance of a stable ERT dose for ≥ 3 months before baseline assessment (at least 80% of the dose specified in the package insert), and an estimated glomerular filtration rate (eGFR) ≥ 30 mL/min/1.73 m^2^ (calculated by the modification of diet in renal disease equation) at screening. In patients receiving an angiotensin-converting enzyme inhibitor, angiotensin II receptor blocker, or renin inhibitor, doses of these drugs had to be stable for ≥ 4 weeks before screening.

Eligible patients were randomly assigned 1.5:1 using interactive response technology (Almac Clinical Technologies, Craigavon, UK) to receive either oral migalastat hydrochloride (150 mg every other day) or continue previous ERT (biweekly infusions of either agalsidase alfa 0.2 mg/kg or agalsidase beta 1.0 mg/kg) during the 18-month open-label treatment period [18]. Patients who completed the open-label comparison period could continue treatment with migalastat (150 mg every other day) for an additional 12 months. During this OLE period, patients in the migalastat group continued to receive migalastat, whereas patients in the ERT group discontinued ERT and switched to migalastat. Patients who completed both parts of the ATTRACT study were eligible for enrollment in the OLE study (AT1001-042, ClinicalTrials.gov, NCT02194985).

### End points

Coprimary end points of the ATTRACT study were the annual rate of change from baseline in eGFR calculated using the chronic kidney disease epidemiology collaboration equation (eGFR_CKD-EPI_) and measured GFR determined by clearance of iohexol (mGFR_iohexol_) [18]. Key secondary end points were changes from baseline in LVMi and composite clinical outcome (renal, cardiac, cerebrovascular events, and death). Other secondary end points were leukocyte α-Gal A activity, plasma lyso-Gb3 level, and safety.

The efficacy analysis was performed in the modified intention-to-treat population, which included all randomly assigned patients who received at least 1 dose of study drug and had baseline and postbaseline GFR measurements, excluding patients with *GLA* mutations later found nonamenable in a new and good laboratory practice-validated HEK assay [13]. The safety population included all randomly assigned patients who received ≥ 1 dose of the study medication.

### Statistical analysis

Leukocyte α-Gal A activity was measured at baseline, months 1 and 3, and every 3 months thereafter up to month 30. Annualized rates of change in eGFR_CKD-EPI_ and mGFR_iohexol_ were analyzed by analysis of covariance, considering the treatment group, sex, age, baseline GFR, and baseline 24-h urine protein excretion as covariates. Descriptive statistics, including least squares mean values and 95% confidence intervals (CIs), were determined with this model. Annualized rates of change of GFR were calculated from the slopes obtained by linear regression. LVMi was evaluated centrally (Cardiocore, Rockville, MD, USA) by 2-dimensional or M-mode echocardiography in a blinded manner every 6 months. The long-term effect of migalastat on LVMi was evaluated by calculating the change from baseline to final assessment in each patient. Composite clinical outcome was evaluated based on the number of patients who experienced a prespecified renal event, cardiac event, cerebrovascular event or who died. The plasma level of lyso-Gb_3_ was quantified by liquid chromatography–mass spectrometry at baseline and months 6, 12, 18, and 30.

Safety was evaluated based on the type, frequency, and severity of adverse events (AEs), as well as changes in vital signs, laboratory data, and physical findings. AEs were coded according to the Medical Dictionary for Regulatory Activities (MedDRA version 8.0 or more recent versions).

## Results

### Patients

The ATTRACT study cohort included 60 patients initially randomly assigned to receive migalastat (*n* = 36) or to continue ERT (*n* = 24) for the first 18-month open-label comparison period. Three patients randomly assigned to the ERT group discontinued the study before the first dose; therefore, 57 patients (36 in the migalastat group and 21 in the ERT group) were included in the primary analysis. These 57 patients were reported to be amenable to migalastat based on the preliminary HEK cell-based assay. A new, good laboratory practice-validated HEK cell-based assay became available during the study and confirmed that 53 of these 57 patients had migalastat-amenable mutations [18].

**Fig. 1:**
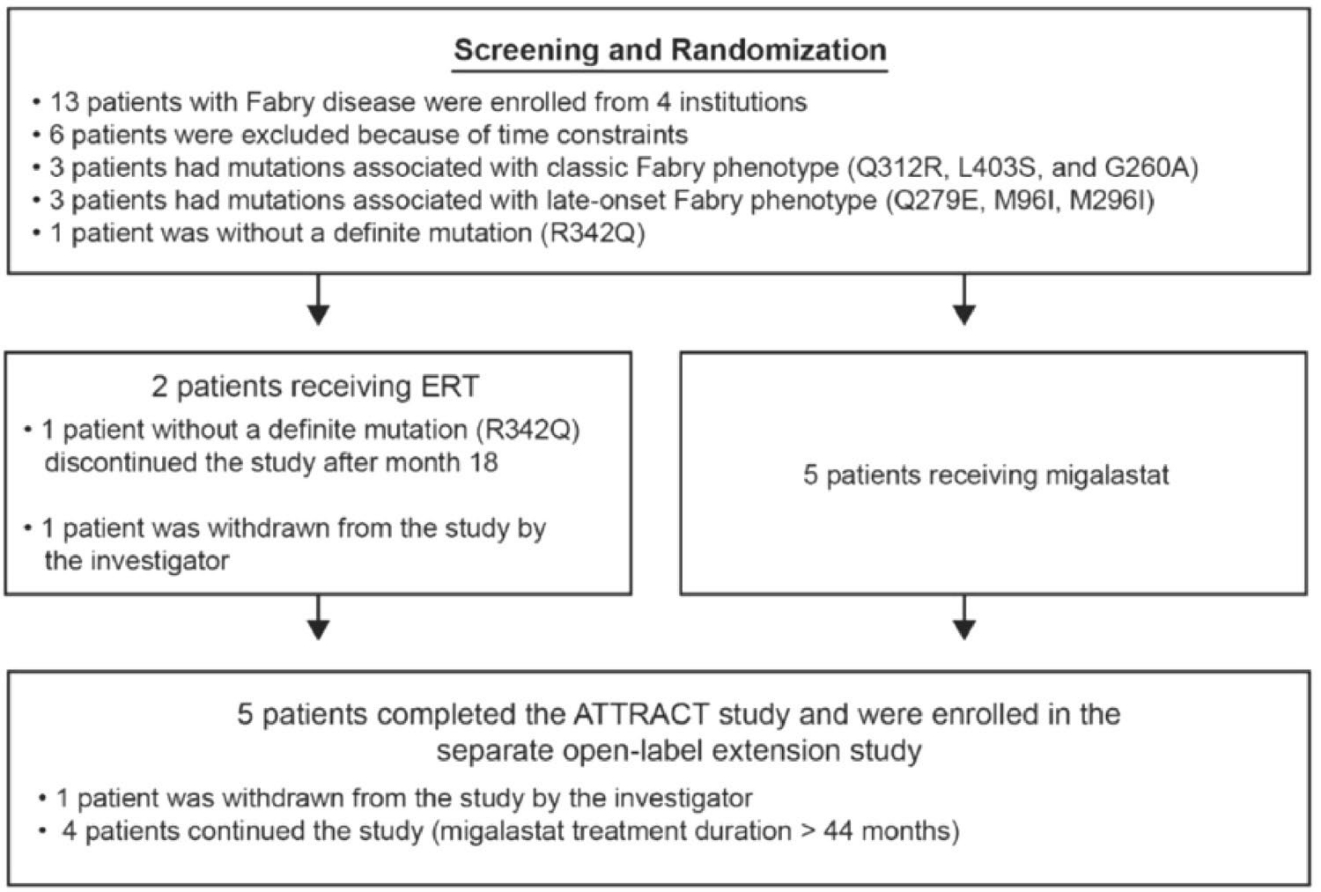
Patient flowchart: ATTRACT study. *ERT* enzyme replacement therapy

The shortage of agalsidase beta in Japan between 2009 and 2012 made it difficult to find patients who met the enrollment criteria for the ATTRACT study (i.e., ≥ 12 months of ERT with stable dosing) [20]. Despite this, 7 patients (> 10% of the ATTRACT population) were enrolled from participating medical institutions throughout Japan. Five patients (3 men and 2 women, Table 1) were randomly assigned to the migalastat group and 2 patients (2 women) were randomly assigned to the ERT group (Fig. 1). Three patients had mutations associated with the classic phenotype of Fabry disease, and 3 had mutations associated with the late-onset phenotype. One patient randomly assigned to the ERT group had nonamenable mutation (R342Q) and was discontinued from the study after month 18; this patient was not included in the efficacy analyses. The other patient randomly assigned to the ERT group was withdrawn by the investigator at completion of the study due to deterioration of chronic heart failure. All 5 patients randomly assigned to the migalastat group completed the 30-month ATTRACT study and were then enrolled in the AT1001-042 OLE study. Of these, 1 patient subsequently was withdrawn from the OLE study by the investigator due to reduced renal function; the other 4 patients continued the OLE study (migalastat treatment duration > 44 months).

**Table 1:** Baseline characteristics of the Japanese patient population and the overall patient population of the ATTRACT study (mITT population)

Item	Japanese patient population			Overall patient population		
	Migalastat (*n* = 5)	ERT (*n* = 1)	All (*n *= 6)	Migalastat (*n* = 34)	ERT (*n* = 18)	All (*n* = 52)
Age, years, mean (SD)	51.8 (4.8)	70.0	54.8 (8.6)	51.2 (13.2)	44.8 (14.7)	49.0 (14.0)
Sex, *n* (%)						
Male	3 (60.0)	0	3 (50.0)	14 (41.2)	8 (44.4)	22 (42.3)
Female	2 (40.0)	1 (100.0)	3 (50.0)	20 (58.8)	10 (55.6)	30 (57.7)
Race, *n* (%)						
White	0	0	0	28 (82.4)	17 (94.4)	45 (86.5)
Asian	5 (100.0)	1 (100.0)	6 (100.0)	5 (14.7)	1 (5.6)	6 (11.5)
Other	0	0	0	1 (2.9)	0	1 (1.9)

*ERT* enzyme replacement therapy, *mITT* modified intention-to-treat population; *SD*, standard deviation

Patients in the mITT population are those with amenable *GLA* mutations who were assessed for baseline and postbaseline efficacy measures

The Japanese patient population was similar to the overall patient population of the ATTRACT study (Table 1), but the mean age was slightly older (55 vs 49 years, respectively). There was an equal number of male and female patients in both patient populations.

### Efficacy

Table 2 shows data on the primary and secondary end points in the Japanese patient population.

**Table 2:** Individual patient characteristics and results for primary and key secondary end points [18-month comparison period + 12-month extension period (30 months of treatment)]

Group	Migalastat–migalastat (*n* = 5)					ERT–migalastat (*n* = 2)	
Subject no.	Subject 1	Subject 2	Subject 3	Subject 4	Subject 5	Subject 6^b^	Subject 7
Genotype	Q312R	L403S	Q279E	M296I	M96I	R342Q	G260A
Age, years	48	56	57	52	46	53	70
Sex	Male	Male	Female	Female	Male	Female	Female
Duration of disease, years	19.74	4.63	4.78	17.31	4.20	10.73	26.82
ACEI/ARB use	Yes	Yes	No	Yes	Yes	No	No
eGFR_CKD-EPI_ (mL/min/1.73 m^2^)							
Baseline	51.33	99.74	97.73	108.34	54.91	106.85	44.83
Change (0–18 months of administration)	–11.93	0	–6.99	–2.14	–2.85	–7.43	4.97
Annualized rate of change (0–18 months of administration)	–6.97	2.25	–4.25	1.53	–1.48	–1.77	1.56
Change (0/18–30 months^a^ of administration)	–17.66	0.58	–8.65	–3.48	–6.26	–	–17.60
Annualized rate of change (0/18–30 months^a^ of administration)	–5.82	0.10	–6.26	–0.73	–1.98	–	–20.26
mGFR_iohexol_ (mL/min/1.73 m^2^)							
Baseline	56.20	99.80	107.60	106.80	52.80	108.00	33.00
Change (0–18 months of administration)	–10.50	–20.00	–25.80	–1.10	–1.70	–4.20	–6.30^c^
Annualized rate of change (0–18 months of administration)	–7.22	–11.40	–16.32	–1.93	–1.11	–4.70	–6.42^c^
Change (0/18–30 months^a^ of administration)	–15.10	–13.10	–0.40	–5.40	–5.60	–	–
Annualized rate of change (0/18–30 months^a^ of administration)	–6.02	–5.85	–0.11	–2.27	–2.68	–	–
Left ventricular mass index (g/m^2^)							
Baseline	102.64	165.73	87.83	74.83	125.63	122.49	85.34
Change (0–18 months of administration)	–7.74	–26.23	1.63	–8.72	–12.59	–23.97	12.54
Change (0/18–30 months^a^ of administration)	–17.96	–24.46	–0.12	–18.86	–5.13	–	13.26
α-Gal A activity in PBMC (nmol/h/mg)							
Baseline	1.69	1.48	22.42	17.61	1.23	11.91	13.64
Change (0–18 months of administration)	6.86	0.74	5.07	13.06	2.25	3.57	–6.31
Change (0/18–30 months^a^ of administration)	3.48	1.02	13.44	15.15	2.86	–	16.98
Plasma lyso-Gb_3_ level (nmol/L)							
Baseline	2.247	11.500	3.220	1.380	14.500	13.13	10.93
Change (0–18 months of administration)	0.767	–1.107	0.277	0.550	–0.200	–2.37	–0.57^c^
Change (0/18–30 months^a^ of administration)	1.050	–2.127	0.967	0.733	–0.833	–	–

*α*-*Gal A* alpha-galactosidase A, *ACEI* angiotensin-converting enzyme inhibitor, *ARB* angiotensin II receptor blocker; *HEK* human embryonic kidney, eGFR_CKD-*EPI*_ estimated glomerular filtration rate calculated by the Chronic Kidney Disease Epidemiology Collaboration equation, *ERT* enzyme replacement therapy, *lyso*-*Gb*_*3*_ globotriaosylsphingosine, *mGFR*_*iohexol*_ estimated glomerular filtration rate determined from clearance of iohexol, *mITT* modified intention-to-treat population, *PBMC* peripheral blood mononuclear cell

^a^0/18–30 months: the change or annualized rate of change from baseline in the migalastat–migalastat group, or the change or annualized rate of change from month 18 in the ERT-migalastat group

^b^The subject was identified as having a *GLA* gene mutation not amenable to migalastat based on the final HEK cell-based assay and was excluded from the mITT population

^c^Month 12

#### Leukocyte α-Gal A activity

Three Japanese men receiving migalastat showed an increase in leukocyte α-Gal A activity from baseline to month 18 (change from baseline: 0.74 nmol/h/mg, 2.25 nmol/h/mg, and 6.86 nmol/h/mg; percentage change: 50%, 183%, and 406%). In all men with amenable mutations in the migalastat group of the ATTRACT study (*n* = 14, including 3 Japanese men), the median change in leukocyte α-Gal A activity during the same period was 6.6 nmol/h/mg. In contrast, patients in the ERT group showed no change in leukocyte α-Gal A activity from baseline (median change, 0.04 nmol/h/mg). Because women express both the mutant and the wild-type forms of α-Gal A, measurement of leukocyte α-Gal A activity in the female patients was considered inappropriate.

#### Renal function

In the overall patient population, migalastat and ERT showed a similar effect on renal function during the 18-month treatment period [17]. The mean annualized rate of change of eGFR_CKD-EPI_ was −0.4 mL/min/1.73 m^2^ in the migalastat group (*n* = 34) and −1.0 mL/min/1.73 m^2^ in the ERT group (*n* = 18). The mean annualized rate of change of mGFR_iohexol_ was −4.4 mL/min/1.73 m^2^ in the migalastat group (*n* = 34) and −3.2 mL/min/1.73 m^2^ in the ERT group (*n* = 18) [18].

**Fig. 2:**
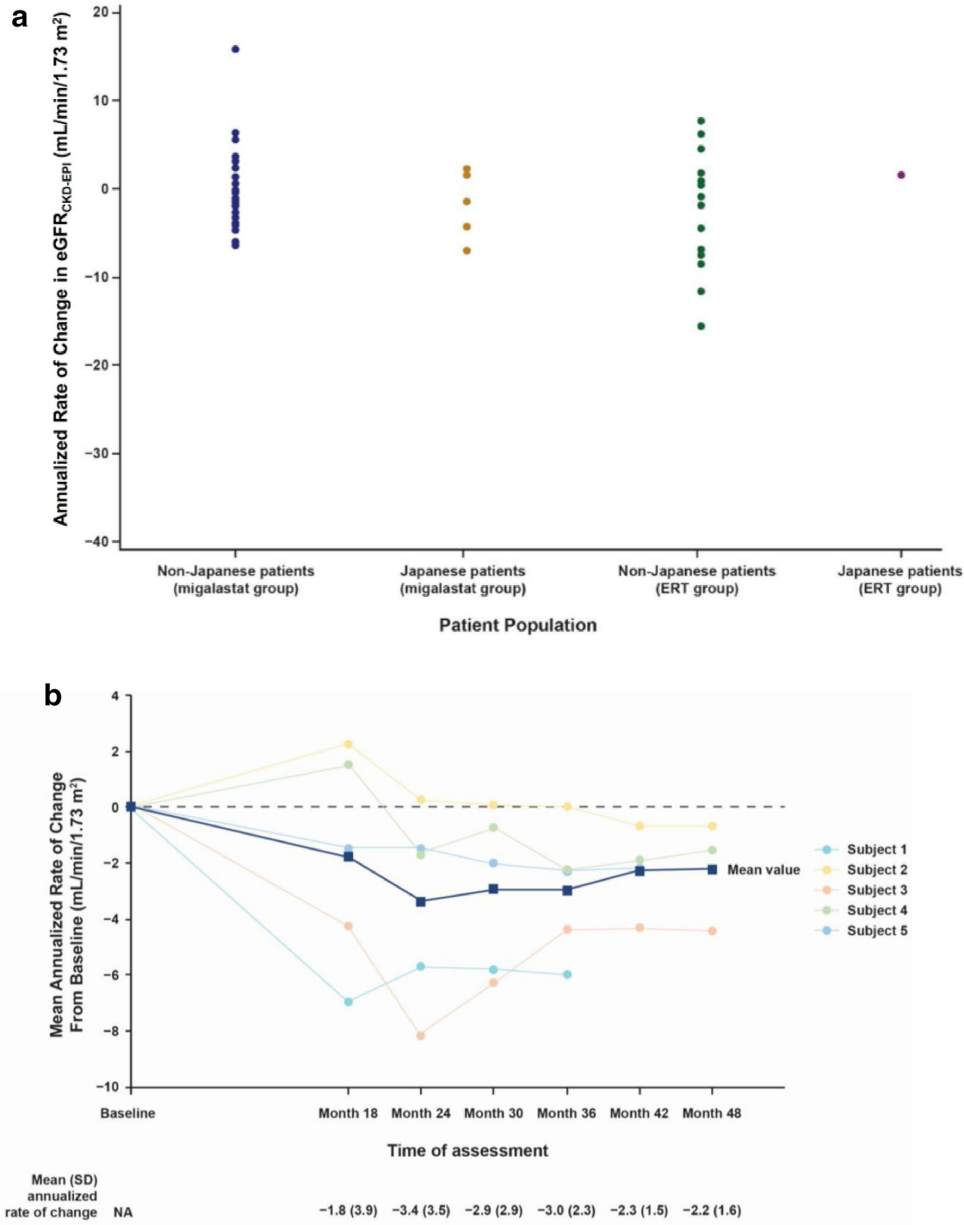

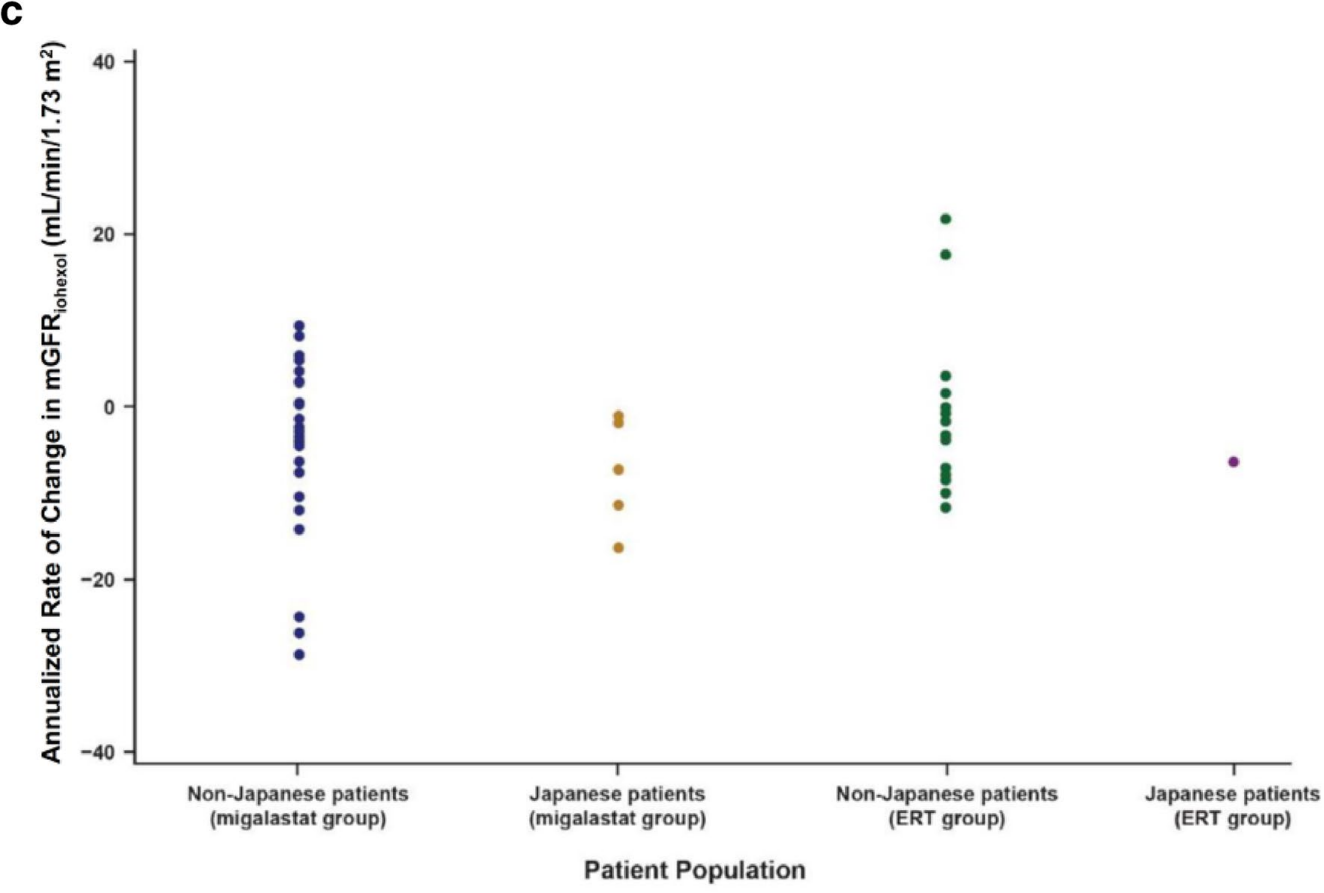
Annualized rate of change in the glomerular filtration rate (mL/min/1.73 m^2^). **a** Annualized rate of change of eGFR_CKD-EPI_ from baseline to month 18. Dots are individual patient data. **b** Annualized rate of change of eGFR_CKD-EPI_ from baseline to month 48 in the 5 Japanese patients randomly assigned to the migalastat group at study entry. Because 1 patient was withdrawn from the study by the investigator at month 30 or thereafter, data for month 42 and month 48 are from the remaining 4 patients. Blue line is mean change from baseline; remaining lines represent individual patient data. **c** Annualized rate of change of mGFR_iohexol_ from baseline to month 18. *eGFR*_*CKD*-*EPI*_ estimated glomerular filtration rate calculated by the Chronic Kidney Disease Epidemiology Collaboration equation, *ERT* enzyme replacement therapy, *mGFR*_*iohexol*_ estimated glomerular filtration rate determined from clearance of iohexol, *N/A* not applicable, *SD* standard deviation

In the Japanese patient subpopulation, the mean annualized rate of change in eGFR_CKD-EPI_ from baseline to month 18 was −1.8 mL/min/1.73 m^2^ for the migalastat group (*n* = 5) and 1.6 mL/min/1.73 m^2^ for the ERT group (*n* = 1) (Fig. 2a). Annualized rates of change in the 5 patients from the migalastat group ranged from −6.97 mL/min/1.73 m^2^ to 2.25 mL/min/1.73 m^2^. The mean annualized rate of change of eGFR_CKD-EPI_ in the Japanese patient population was within the 95% CI of the least squares mean for the overall patient population.

In the 5 patients who received migalastat from months 18 to 30 after completion of the open-label comparison period, renal function was stable through month 30 [mean ± standard deviation (SD) annualized rate of change in eGFR_CKD-EPI_ from baseline to month 30: −2.94 ± 2.93 mL/min/1.73 m^2^]. In addition, renal function was stable up to month 48 in the AT1001-042 OLE study (Fig. 2b).

In the Japanese patient subpopulation, the mean annualized rate of change in mGFR_iohexol_ from baseline to month 18 was −7.6 mL/min/1.73 m^2^ for the migalastat group (*n* = 5) and −6.4 mL/min/1.73 m^2^ for the ERT group (n = 1). In the 5 patients from the migalastat group, the annualized rate of change in mGFR_iohexol_ ranged from −16.3 mL/min/1.73 m^2^ to −1.1 mL/min/1.73 m^2^ (Fig. 2c). This range was within the 95% CI of the least squares mean for the overall patient population. In addition, mGFR_iohexol_ was stable through month 30 (mean ± SD annualized rate of change of mGFR_iohexol_ from baseline to month 30, −3.39 ± 2.52 mL/min/1.73 m^2^).

#### Cardiac function

In the overall patient population, a significant reduction in the mean of LVMi from baseline to month 18 was noted among patients who switched from ERT to migalastat at the beginning of the study (−6.6 g/m^2^; 95% CI −11.0 to −2.2; *n* = 33). No significant change was noted in patients who continued ERT (−2.0 g/m^2^; 95% CI −11.0 to 7.0; *n* = 16) [17]. Patients who received migalastat and had baseline left ventricular hypertrophy (*n* = 13) showed the largest changes in LVMi [18].

In the Japanese patient subpopulation, the mean LVMi was reduced from baseline to month 18 in the migalastat group (mean change, −13.8 g/m^2^; *n* = 5). The change in LVMi in the Japanese patients who received migalastat was greater than in the overall ATTRACT patient population (−13.8 g/m^2^ vs −6.6 g/m^2^). Mean baseline LVMi was higher in the Japanese patient population than in the overall patient population (111.3 g/m^2^ vs 97.5 g/m^2^). As in the overall patient population, Japanese patients who had baseline left ventricular hypertrophy showed a greater decrease of LVMi from baseline to month 18 (−19.4 g/m^2^).

**Fig. 3:**
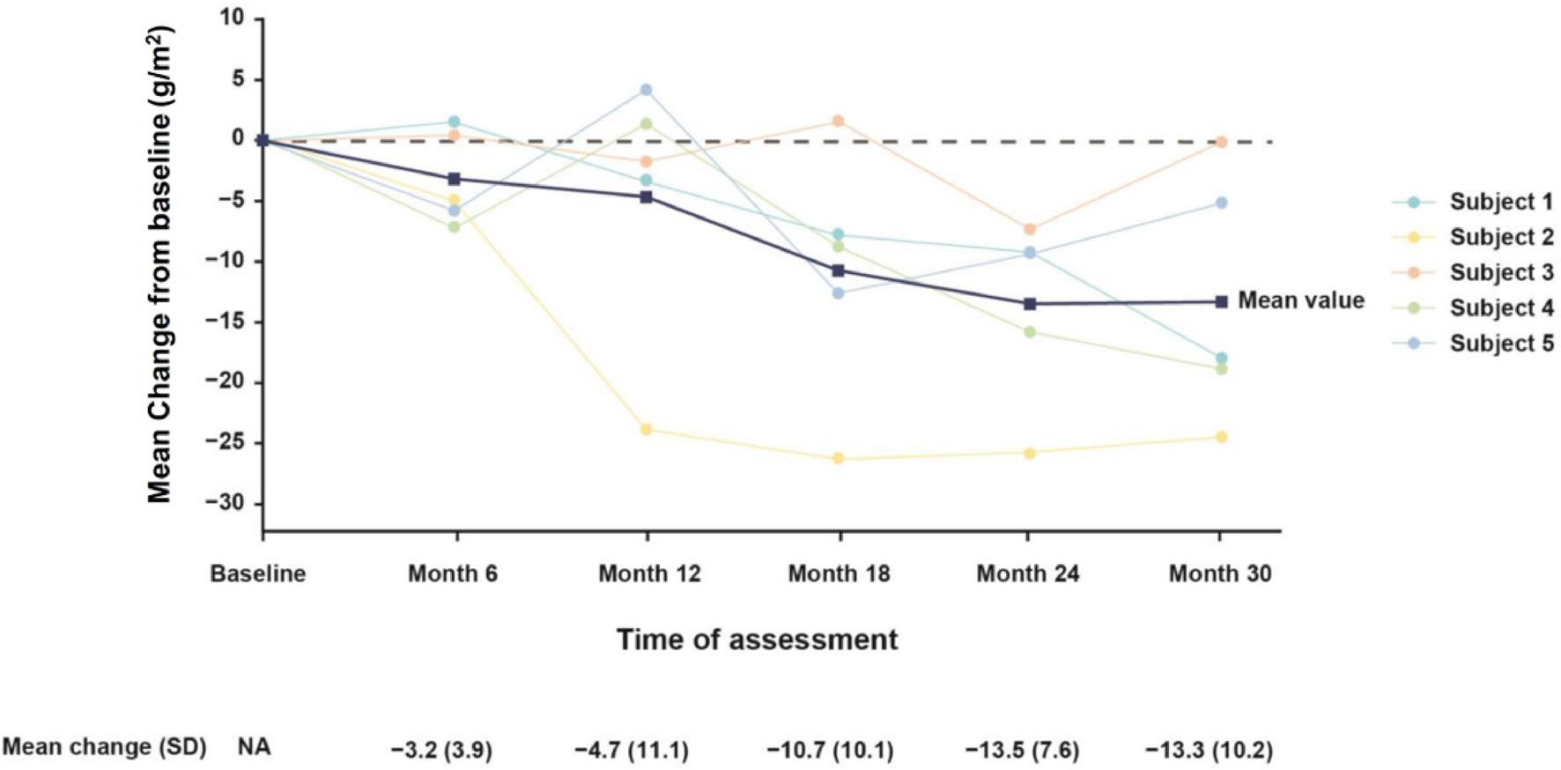
Change in LVMi (g/m^2^) from baseline to month 30 in Japanese patients who received migalastat. LVMi data are from 5 Japanese patients who were randomly assigned to migalastat in the ATTRACT study and then enrolled in AT1001-042 OLE study. Blue line is mean change from baseline; remaining lines represent individual patient data. *LVMi* left ventricular mass index, *N/A* not applicable, *OLE* open-label extension, *SD* standard deviation

In the migalastat group (*n* = 5), the decrease in LVMi persisted to month 30 (Fig. 3). In the OLE study AT1001-042, the mean change of LVMi from baseline to month 42 was −27.2 g/m^2^ in 2 patients with echocardiographic data.

#### Composite clinical outcomes

In the overall patient population, the occurrence of combined clinical outcome (renal events, cardiac events, cerebrovascular events, and death) through month 18 was 29% in the migalastat group and 44% in the ERT group (*p* = 0.36). No deaths were reported during the 18-month open-label comparison period [18].

Among the Japanese patients, a renal event (i.e., > 33% increase of 24-h urinary protein excretion compared with baseline and 24-h urinary protein excretion ≥ 300 mg) occurred in 1 of 5 patients in the migalastat group during the 18-month open-label comparison period, and a cardiac event (i.e., New York Heart Association class III/IV congestive heart failure) occurred in the 1 patient in the ERT group (Table 3).

**Table 3:** Number of events attributable to Fabry disease up to month 18 in the Japanese patient population and the overall patient population (mITT population) of the ATTRACT study

Event, *n* (%)	Japanese		Overall	
	Migalastat (*n* = 5)	ERT (*n* = 1)	Migalastat (*n* = 34)	ERT (*n* = 18)
Renal	1 (20.0)^a^	0	8 (23.5)	6 (33.3)
Cardiac	0	1 (100.0)^b^	2 (5.9)	3 (16.7)
Cerebrovascular	0	0	0	1 (5.6)
Death	0	0	0	0
Total	1 (20.0)	1 (100.0)	10 (29.4)	8 (44.4)

*ERT* enzyme replacement therapy, *mITT* modified intention-to-treat population

Patients in the mITT population are those with amenable *GLA* mutations who were assessed for baseline and postbaseline efficacy measures

^a^> 33% increase of 24-h urinary protein excretion compared with baseline and 24-h urinary protein excretion ≥ 300 mg

^b^New York Heart Association class III/IV congestive heart failure

#### Plasma lyso-Gb_3_ level

In patients from the overall population with amenable *GLA* mutations, the plasma lyso-Gb_3_ level remained low and stable after switching from ERT to migalastat [18]. In the Japanese patients who received migalastat (*n* = 5), the mean baseline plasma lyso-Gb_3_ level was 6.57 nmol/L. As in the overall patient population, plasma lyso-Gb_3_ levels remained low and stable through month 18 during migalastat treatment in the Japanese patients. The mean change of plasma lyso-Gb_3_ from baseline to month 18 was 0.06 nmol/L, which was within the 95% CI of the mean change in the overall patient population (95% CI −0.30 to 3.76).

In these 5 Japanese patients in the migalastat group, the mean plasma lyso-Gb_3_ level remained low and stable through month 30 (mean change from baseline to month 30, −0.04 nmol/L; 95% CI −1.80 to 1.70). At month 30, the plasma lyso-Gb_3_ level ranged from 3.30 to 13.67 nmol/L for men and from 2.11 to 4.19 nmol/L for women.

### Safety

In the overall patient population, the frequency of AEs was 94% in the migalastat group and 95% in the ERT group [17]. The most frequently reported AEs in the migalastat group were nasopharyngitis (33%) and headache (25%). Most were mild or moderate. None of the patients discontinued the study because of AEs, and no treatment-related deaths or serious AEs were reported [18].

In the Japanese patient subpopulation, the frequency of AEs from baseline to month 30 was 100% in the migalastat group (5 of 5 patients) (Table 4). All AEs were mild or moderate. Nasopharyngitis was the only AE that occurred in 2 or more patients (*n* = 4, 80%). No safety concerns were reported in the OLE study AT1001-042. During the OLE period, nasopharyngitis was the only AE that occurred in 2 or more patients (*n* = 2, 40%). None of the Japanese patients experienced headache at any point during the 2 studies. No clinically meaningful changes in vital signs, laboratory data, or physical findings were noted in the Japanese patient subpopulation.

**Table 4:** Adverse events in the Japanese patients randomly assigned to the migalastat group (*n* = 5)

	Month 0–30 (ATTRACT)	After month 30 (AT1001-042 OLE study)
Number of AEs	34	15
Number of patients who experienced AEs, *n* (%)	5 (100.0)	4 (80.0)
The most frequently reported AEs (reported in 2 or more Japanese patients), *n* (%)		
Nasopharyngitis	4 (80.0)	2 (40.0)

*AE* adverse event, *OLE* open-label extension

## Discussion

Migalastat is an oral agent that acts as a pharmacologic chaperone for α-Gal A. In previous studies, migalastat provided clinical benefit with good tolerability in patients with Fabry disease and amenable *GLA* mutations [18, 21]. In the phase III FACETS study, migalastat was administered to patients with Fabry disease who had not received ERT. Results showed a decrease in renal interstitial capillary GL-3 inclusions and plasma lyso-Gb_3_ level due to migalastat treatment, along with stabilization of renal function, a decrease in cardiac mass, and improvement in gastrointestinal symptoms [21]. In the phase III ATTRACT study, migalastat is a treatment option for ERT-experienced patients [18].

No racial differences have been reported with regard to Fabry disease, and its clinical phenotypes are likely similar among races [2, 6, 22]. Therefore, it is meaningful that this subanalysis of Japanese patients showed that the efficacy of migalastat was not affected by race; there were also no unexpected safety concerns. The pharmacokinetics of migalastat was similar in the Japanese patients and the overall ATTRACT population, suggesting that migalastat’s therapeutic efficacy is not affected by intrinsic factors or race [17]. In addition, efficacy and safety of migalastat were comparable in the Japanese and the overall patient populations.

Regarding the pharmacodynamic effects of migalastat, male Japanese patients with Fabry disease showed an increase in leukocyte α-Gal A activity, and the median change was within the range for the overall ATTRACT population. In Japanese patients with amenable mutations, migalastat treatment also stabilized renal function and decreased LVMi, as observed in the overall patient population. In addition, the plasma lyso-Gb_3_ level remained low and stable throughout migalastat treatment in the Japanese patients and in the overall ATTRACT population. Additionally, individual patient data in migalastat group (Table 2) showed that eGFR range was relatively wide which might be due to either varied eGFR level of patients at baseline or concomitant treatment such as ACEI/ARB received by patients. However, given the small sample size in this study, the definitive cause could not be determined.

In the AT1001-042 OLE study, the beneficial effects of migalastat on renal function and LVMi were maintained in the Japanese patients through month 48 and month 42, respectively. In addition to demonstrating efficacy, migalastat was also safe and well tolerated by Japanese patients. The types and frequencies of AEs were similar in both the Japanese and the overall patient populations.

Only a few prospective studies have been performed in Japanese patients with Fabry disease, highlighting the importance of the current findings, despite the small sample size. A recently reported observational study conducted in 36 previously untreated Japanese patients showed that ERT can stabilize renal function and prevent deterioration of cardiac function [6]. The patients in this recent study were relatively young (mean age 27 and 45 years for men and women, respectively), whereas the Japanese patients in the ATTRACT study were older (mean age 55 years), had previously been treated with ERT for 12 months or more before enrollment, and had a high disease burden. Use of migalastat achieved favorable outcomes in patients previously treated with ERT in the ATTRACT study, which included an open-label comparison with ERT and an OLE period [18]. Therefore, these findings are important when considering treatment options for Japanese patients with Fabry disease.

The ATTRACT study was characterized by a prospective design and by enrolling typical Japanese patients with Fabry disease. However, given the small sample size of the Japanese patient population, the results should be interpreted with caution when considering migalastat therapy.

With the caveat of the aforementioned study limitations, migalastat seems effective for Japanese patients with Fabry disease who have amenable *GLA* mutations. In addition, migalastat demonstrated similar efficacy and safety in Japanese patients with Fabry disease who had previously been treated with ERT compared with the overall ATTRACT patient population. Therefore, our findings confirm the pharmacologic actions and efficacy of migalastat in Japanese patients.
